# HuntMi: an efficient and taxon-specific approach in pre-miRNA identification

**DOI:** 10.1186/1471-2105-14-83

**Published:** 2013-03-05

**Authors:** Adam Gudyś, Michał Wojciech Szcześniak, Marek Sikora, Izabela Makałowska

**Affiliations:** 1 Institute of Informatics, Faculty Of Automatic Control, Electronics And Computer Science, Silesian University of Technology, Gliwice, Poland; 2 Laboratory of Bioinformatics, Faculty of Biology, Adam Mickiewicz University, Poznan, Poland; 3 Institute of Innovative Technologies EMAG, Katowice, Poland

**Keywords:** MicroRNA, Random forest, Imbalanced learning, Genome analysis

## Abstract

**Background:**

Machine learning techniques are known to be a powerful way of distinguishing microRNA hairpins from pseudo hairpins and have been applied in a number of recognised miRNA search tools. However, many current methods based on machine learning suffer from some drawbacks, including not addressing the class imbalance problem properly. It may lead to overlearning the majority class and/or incorrect assessment of classification performance. Moreover, those tools are effective for a narrow range of species, usually the model ones. This study aims at improving performance of miRNA classification procedure, extending its usability and reducing computational time.

**Results:**

We present HuntMi, a stand-alone machine learning miRNA classification tool. We developed a novel method of dealing with the class imbalance problem called ROC-select, which is based on thresholding score function produced by traditional classifiers. We also introduced new features to the data representation. Several classification algorithms in combination with ROC-select were tested and random forest was selected for the best balance between sensitivity and specificity. Reliable assessment of classification performance is guaranteed by using large, strongly imbalanced, and taxon-specific datasets in 10-fold cross-validation procedure. As a result, HuntMi achieves a considerably better performance than any other miRNA classification tool and can be applied in miRNA search experiments in a wide range of species.

**Conclusions:**

Our results indicate that HuntMi represents an effective and flexible tool for identification of new microRNAs in animals, plants and viruses. ROC-select strategy proves to be superior to other methods of dealing with class imbalance problem and can possibly be used in other machine learning classification tasks. The HuntMi software as well as datasets used in the research are freely available at http://lemur.amu.edu.pl/share/HuntMi/.

## Background

MicroRNAs (miRNAs) are ∼21 bases long RNAs that post-transcriptionally control multiple biological processes, such as development, hematopoiesis, apoptosis and cell proliferation [1]. Mature miRNAs are derived from longer precursors called pre-miRNAs that fold into hairpin structures containing one or more mature miRNAs in one or both arms [2]. Their biogenesis is highly regulated at both transcriptional and post-transcriptional levels [3], and disregulation of miRNAs is linked to various human diseases, including cancer [4].

Identification of miRNA is a challenging task that allows us to better understand post-transcriptional regulation of gene expression. In last ten years a number of experimental and computational approaches were proposed to deal with the problem. However, experimental approaches, including direct cloning and Northern blot, are usually able to detect only abundant miRNAs. MicroRNAs that are expressed at very low levels or in a tissue- or stage-specific manner, often remain undetected. These problems are partially addressed by applying the deep-sequencing techniques that nevertheless require extensive computational analyses to distinguish miRNAs from other non-coding RNAs or products of RNA degradation [5].

Computational approaches in miRNA search can be homology-based, take advantage of machine learning methods, or use both of these. Homology-based approaches rely on conservation of sequences, secondary structures or miRNA target sites (e.g. RNAmicro [6], MIRcheck [7]). As a result, these methods are not suitable for detection of lineage- or species-specific miRNAs and miRNAs that evolve rapidly. Moreover, they are strongly limited by the current data and performance of available computational methods, including alignment algorithms [8]. Another problem is that there are as many as ∼11 million sequences that can fold into miRNA-like hairpins in the human genome [9], some of which originate from functional, non-miRNA loci. It is therefore no surprise that a large number of hairpins that are conserved between species could be mistakenly classified as miRNAs. Nevertheless, homology search has been successfully applied in many miRNA gene predictions, in both animals and plants [10,11].

In some approaches, e.g. PalGrade [12] or miRDeep [5], experimental and computational procedures are combined. However, as mentioned above, experimental methods can not easily detect low-expression or tissue-specific miRNAs and/or they have to meet computational challenges, as in the case of deep sequencing technology. miRDeep, for instance, aligns deep sequencing reads to the genome and selects the regions that can form a hairpin structure. Then, using a probabilistic model, the hairpins are scored based on the compatibility of the position and frequency of sequenced reads with the secondary structure of the pre-miRNA. This method achieves high specificity at the cost of relatively low sensitivity.

Machine learning methods are amongst the most popular ways of miRNA identification nowadays. They share the same overall strategy. First, the features of primary sequence and secondary structure are extracted from known miRNAs (positive set) and non-miRNA sequences (negative set). Then, the features are used to construct a model which serves to classify candidate sequences as real pre-miRNAs or pseudo pre-miRNAs. There are several machine learning methods that have been applied in the field of miRNA identification. These include hidden Markov models (HMM) [13], random forest [14] and naïve Bayes classifier [15]. Support vector machine, however, seems to be the most popular framework nowadays and has been used in a number of well recognised tools. For instance, Triplet-SVM [16] classifies real human pre-miRNAs and pseudo pre-miRNAs using 32 structure- and sequence-derived features that refer to the dot-bracket representation of the secondary structure i.e. it considers the frequencies of triplets, such as "A(((" and "U.(.", consisting of the secondary structure of three adjacent nucleotides and the nucleotide in the middle. miPred [8] classifies human pre-miRNAs from pseudo hairpins represented by twenty nine folding features, using SVM-based approach. The features were evaluated with the F scores F1 and F2 on the class-conditional distributions to assess their discriminative power. Strongly correlated attributes were rejected. microPred [17] presents nineteen new features along with twenty nine taken from miPred. After feature selection, twenty one attributes were used to train the classifier. The improved feature selection approach and addressing the class imbalance problem resulted in high sensitivity and specificity of the method.

However, the existing machine learning approaches suffer from some drawbacks. First of all, they often make structural assumptions concerning stem length, loop size and numbers as well as a minimum free energy (MFE). Secondly, most of existing miRNA classifiers work well on data from model species and closely related ones; the classifiers trained on human data best fit the miRNA identification problem in human and other primates but perform unsatisfactorily when applied to, for example, invertebrates. Finally, the imbalance problem between the positive and negative classes is usually not addressed properly, while this is a crucial issue, as the number of microRNAs throughout a genome is much lower than the number of non-microRNAs (e.g. ∼1 400 miRNAs vs. ∼11 million pseudo hairpins in *H. sapiens*). The resulting difference in misclassification costs of positive and negative classes requires special techniques of learning from imbalanced data as well as a proper assessment metrics. Moreover, in order to accurately judge classifier performance in real-life applications, the problem of imbalance should be reflected in the testing datasets.

In this study we addressed all these issues. We made no preliminary assumptions about miRNA structure and carefully took into account class imbalance problem. We implemented a procedure of thresholding score function produced by traditional classifiers and called it ROC-select. This strategy turned out to be superior to other imbalance-suited techniques in miRNA classification. From all classifiers for which ROC-select procedure was applied we chose random forest as it yields the best balance between sensitivity and specificity. Regarding the data representation, we introduced seven new features and show that they further improve the classification performance. In the experiments we considered large and strongly imbalanced up-to-date sets of positive and negative examples, paying much attention to the data quality. The tests were performed using stratified 10-fold cross-validation (CV) giving reliable estimates of classification performance. Finally, we show that the method outperforms the existing miRNA classification tools, including microPred, without compromising the computational time.

Our miRNA classification method is freely available as a framework called HuntMi. HuntMi comes with trained models for animals, plants, viruses and separately for *H. sapiens* and *A. thaliana*. As a result, the tool can be used in miRNA classification experiments in a wide range of species. The user can use built-in models in the experiments or train new models using custom datasets prior to classification.

## Methods

### Datasets

In order to create positive sets, we retrieved all pre-miRNAs from miRBase release 17 [18] and filtered out the sequences lacking experimental confirmation. By using evidence-supported miRNAs only, we minimize the chance of introducing false positives into the set. The sequences were divided into five groups: *H. sapiens*, *A. thaliana*, animals, plants, and viruses.

Negative sets were extracted from genomes and mRNAs of ten animal and seven plant species as well as twenty nine viruses (Additional file 1: Table S1). Additional sets were prepared for *H. sapiens* and *A. thaliana*. Start positions were randomly selected, whereas end positions were calculated so that the sequence length distribution in the resulting negative dataset is the same as in the corresponding positive one. With this approach, the classifier achieves better performance when applied in real-life experiments, where miRNA candidates tend to have lengths similar to those of known miRNAs. Finally, in order to remove known miRNAs together with similar sequences that possibly represent unknown homologs of miRNAs, we ran BLASTN search against miRBase hairpins and filtered out sequences that produced E-value of 10^−2^ or lower. 96.17% of negative sequences prepared in this way possess structural features of real pre-microRNAs, including the minimum free energy below -0.05 (normalised to the sequence length) and number of pairings in the stem above 0.15 (also normalised to the length). At the same time these criteria are met by 97.61% of hairpins stored in miRBase.

Positive and negative sequences from the analysed species were gathered to form complete datasets that correspond to miRNA classification problem in the taxa. They will be referred to as *human*, *arabidopsis*, *animal*, *plant* and *virus* (Table 1). In addition, we used the dataset from microPred. It contains 691 non-redundant human pre-miRNAs from miRBase release 12, 754 non-miRNA ncRNA, 8 494 pseudo hairpins and is denoted as *microPred*.

**Table 1:** Datasets characteristics

**Name**	**#Positives**	**#Negatives**	**Imbalance**
*human*	1 406	81 228	57.8
*arabidopsis*	231	28 359	122.8
*animal*	7 053	218 154	30.9
*plant*	2 172	114 929	52.9
*virus*	237	839	3.5
*microPred*	691	9 248	13.4

Characteristics of biological datasets used in the experiments. Imbalance is defined as a ratio of #Negatives to #Positives. We limited dataset imbalance to several tens for practical reasons even though proportions of miRNAs to non-miRNAs in genomes are more extreme. In the case of virus dataset the imbalance is exceptionally low as we wanted to know how methods perform on moderately imbalanced problems. In addition, it is difficult to create representative dataset for viruses as their genomes differ significantly in sizes and most of them do not contain miRNAs.

### Features

The twenty one features selected by [17] were used as a base representation in the experiments. Thus, we employed microPred scripts for extracting necessary attributes. In the case of *microPred* dataset we took precalculated features from webpage to make our results comparable with the existing research (some of the features are calculated using randomly generated sequences).

Beside twenty one microPred features, we calculated seven additional sequence- and structure-related attributes. First, we considered the frequencies of secondary structure triplets composed of three adjacent nucleotides and the middle nucleotide. We chose four of them that were shown to have the highest information gain [19]: "A(((", "U(((", "G(((", and "C(((", referred to as *tri_A*, *tri_U*, *tri_G*, and *tri_C*, respectively. The remaining features are: the maximal length of the amino acid string without stop codons found in three reading frames: *orf*; the cumulative size of internal loops found in the secondary structure: *loops*; a percentage of low complexity regions detected in the sequence using Dustmasker: *dm* (all Dustmasker settings were set to default except for score threshold for subwindows set from 20 to 15).

### Imbalanced learning

Extensive research on imbalanced data classification has proven that standard machine learning techniques often overlearn a majority class sacrificing minority examples [20]. Therefore, special approaches for imbalanced problems have been developed. They can be divided into sampling methods, cost-sensitive learning, kernel methods, active learning and others [21]. microPred authors carried out exhaustive study of how several classification strategies from above perform in a microRNA prediction task [17]. They used standard support vector machine as a base classifier and combined it with random over/under-sampling, SMOTE (which is also a representative of sampling methods) and multi-classifier system. They additionally tested cost-sensitive SVM modifications like zSVM and DEC (different error costs), finding SMOTE to be the best strategy. In the research, geometric mean (*G*_*m*_) of classification sensitivity (SE) and specificity (SP) was used as an assessment metric. *G*_*m*_ is common in imbalanced learning problems, including miRNA identification, as it takes into account unequal misclassification costs. Therefore, we also decided to use *G*_*m*_ in HuntMi study.

Our approach to microRNA prediction relies on the fact that classification with unequal costs is equivalent to thresholding conditional class probabilities at arbitrary quantiles [22]. Many classifiers provide continuous score function *s*(*x*) describing degree of a membership of instance *x* to particular class. Ideally, such a function estimates perfectly a class conditional probability *P*(*c*|*x*) and is denoted as well-calibrated score function [23]. In reality, classifiers produce scores which are often not calibrated [22] thus a lot of algorithms for calibrating them have been developed [23]. In addition, many meta learning techniques like bagging or classifier ensembles can be employed to produce score function on the basis of class labels alone [24]. As long as scoring function ranks instances properly, that is *s*(*x*)<*s*(*y*)⇔*P*(*c*|*x*)<*P*(*c*|*y*), one can successfully use *s*(*x*) directly to classify instances with unequal costs.

Our method combines the idea of thresholding classifier score function with receiver operating characteristics (ROC) [25]. For each threshold value *T* established at *s*(*x*) function, a point in a ROC space can be generated. Varying *T* from −*∞* to +*∞* produces entire ROC curve. One can select a point on it with highest evaluation metric (*G*_*m*_ in the case) and read corresponding *T* value. In real applications ROC curves are generated by simply sorting elements of dataset by *s*(*x*) values and updating true positive (*TP*) and false positive (*FP*) statistics for consecutive points. In order to prevent threshold selection procedure from overfitting towards training data, a separate set should be used for constructing ROC curve. Hence, an internal cross-validation with *k*_1_ folds is employed for this purpose. As we are not interested in variance, ROC curves are averaged in a straightforward way - instances from all tuning folds together with assigned *s*(*x*) values are gathered in a single set on which ROC generation procedure is applied [25]. Threshold leading to the highest value of evaluation metric is stored and used for classification of unknown instances. The threshold selection procedure described above will be referred to as ROC-select.

In the research we apply ROC-select only on classifiers directly providing scoring function, no meta learning techniques were examined. These classifiers are naïve Bayes [26], multilayer perceptron [27], support vector machine [28] and random forest [29]. We used radial basis function as an SVM kernel as it is known to produce best classification results in wide range of applications [30]. In order to compare proposed strategy with other methods, we additionally tested SMOTE filter [31] combined with SVM as it gave best results in microPred experiments and a novel method of asymmetric partial least squares classification (APLSC), which came out to be superior to other strategies on several strongly imbalanced datasets [32].

### Parameter selection and complexity analysis

In many studies including microRNA prediction, classifier parameters are selected in order to obtain best possible results for a particular domain. Hence, we decided to place parameter tuning phase in our pipeline as a preceding step for threshold selection. Parameter selection is also done with an internal cross-validation with a number of folds equal to *k*_2_ and is straightforward. At first, a search space is defined by specifying a number of discrete values for each parameter to be tuned. Then, full cross-validation procedure is performed for each point in that space. Combination of parameter values leading to the highest average evaluation metric (*G*_*m*_) is stored and used in threshold selection and, finally, for classification of unknown instances.

Let us denote number of points in the parameter space to be examined as *λ*. In addition, let *L*(*n*) and *T*(*n*) indicate time complexities of training and testing procedures for given classifier with respect to the dataset size *n*. ROC-select and parameter tuning are performed in $O(k_{1}(L(n(k_{1}-1)/k_{1})+T(n/k_{1}))+n\log n)$ and *O*(*λk*_2_(*L*(*n*(*k*_2_−1)/*k*_2_)+*T*(*n*/*k*_2_))) time, respectively. As (*k*−1)/*k*<1 entire procedure is bounded by expression $O((k_{1}+\lambda k_{2})L(n)+k_{1}T(n/k_{1})+\lambda k_{2}T(n/k_{2})+n\log n)$.

### Experimental setting

All classification experiments were carried out using stratified 10-fold CV, hence distributions of testing samples are exactly the same as for the entire datasets. Taking into account strong imbalance of examined sets, obtained results approximate well the expected performance of a classifier in practical applications. Additionally, 10-fold CV was proven to be the best method of model evaluation in terms of bias and variance [33].

The detailed configuration of examined classifiers together with parameter values tested in a tuning phase are listed below (number of points in a parameter space for tuning phase given in parentheses). Parameters not mentioned here remained default. 

•naïve Bayes: kernel estimation turned on,

•multilayer perceptron: validation set size *V*=20*%*, validation threshold *E*=50, learning rate *η*=0.1,0.2,…,0.5, momentum *μ*=0.1,0.2,…,0.5 (*λ*=25),

•SVM: feature normalization turned on, cost *C*=10^−2^,10^−1^,…,10^2^, exponent in radial basis kernel *γ*=2^−2^,2^−1^,…,2^2^ (*λ*=25),

•random forest: number of trees *i*=10,21,…,219 (*λ*=20),

•APLSC: number of dimensions *d*=5,10,15,20 (*λ*=4).

Preliminary experiments on naïve Bayes classifier confirmed that kernel estimation improves classification results, so this feature was turned on. Validation threshold parameter in a multilayer perceptron indicates how many times in a row the validation set error can increase before training is terminated. Early tests showed that introducing validation with this stop condition does not influence classification results but significantly reduces training time, therefore we decided to use it in our research. SMOTE filter was configured to balance positive and negative sets perfectly. SVM parameters in SMOTE + SVM combination were tuned with a wider range of values, that is *C*=10^−2^,10^−1^,...,10^3^, and *γ*=2^−2^,2^−1^,…,2^4^ (*λ*=42). Authors of microPred used a more exhaustive scanning strategy, however it is inapplicable for larger problems because of computational overhead. Hence, we limited search space to cover parameter values selected most commonly in preliminary experiments. Geometric mean (*G*_*m*_) was chosen as an evaluation metric to be maximised. Numbers of folds, *k*_1_ and *k*_2_, were set to 10 and 5, respectively. We decided to use 5-fold CV in the parameter tuning because it allowed us to reduce times of analyses with respect to 10-fold CV almost by half (parameter tuning dominates over other stages in terms of computation time), rendering slightly inferior results [33]. This approach follows microPred, which also used 5-fold CV for parameter tuning.

ROC-Select strategy described in the paper was prepared as a plug-in to Weka [34] package which had been chosen as the basic environment for all classification experiments. It provided us with implementations of naïve Bayes, multilayer perceptron, random forest and SMOTE filter. Weka interface for LibSVM was used for support vector machine experiments. The original APLSC code written in MATLAB was wrapped in Java class and also attached to Weka as a plug-in.

## Results and discussion

### Threshold selection

The first step of the experiments was to check how the threshold selection strategy influences classification results. For each classifier undergoing ROC-select procedure four tests were carried out: no selection (I), threshold selection only (II), parameter selection only (III), both parameter and threshold selection (IV). Relative *G*_*m*_ changes of variants II, III and IV with respect to the variant I were calculated and averaged over all datasets beside *microPred* (Table 2). As one can see, applying threshold selection procedure leads to significant improvement in *G*_*m*_ values. The exception is naïve Bayes for which the gain is moderate. This can be explained by intrinsic resistance of naïve Bayes to the class imbalance problem - it performed well without applying ROC-select. In the case of naïve Bayes no parameters were tuned, thus variants III and IV are the same as I and II, respectively. In other cases the best results were obtained with combination of parameter and threshold tuning. It is important to note that variant II overtakes relevantly variant III. This confirms that standard machine learning techniques are not suited for imbalanced datasets and adjusting classifier parameters can reduce the problem of overlearning majority class only by a small marigin. To achieve best possible performance, classifiers suited for imbalanced problems (SMOTE + SVM and APLSC) were always tested with parameter tuning turned on (variant III). For computational reasons we decided to limit parameter space from 42 points to 25 while running SMOTE + SVM on *animal* set (same points as in SVM and ROC-select combination were used).

**Table 2:** Relative gains in classification results

**Classifier**	**Threshold** **selection (II)**	**Parameter** **selection (III)**	**Parameter** + **threshold** **selection (IV)**
Naïve Bayes	1.11	0.00	1.11
Perceptron	7.70	0.26	7.76
SVM	10.11	1.89	10.29
Random forest	6.95	1.55	9.30

Relative percentage gains in *G*_*m*_ obtained by applying parameter and/or threshold selection on different classifiers averaged over all datasets.

Absolute values of sensitivity, specificity and *G*_*m*_ for particular classifiers and datasets are given in Table 3. As applying ROC-select procedure improved performance much more relevantly than parameter tuning, only results for variants III and IV are presented. The general observation is that traditional classification algorithms at default threshold (variant III) clearly overlearn majority class and lose with SMOTE + SVM and APLSC in terms of *G*_*m*_. The greater class imbalance, the more visible is this regularity. For instance in the case of *virus* dataset, which is only slightly imbalanced, traditional algorithms perform almost as good as imbalance-suited methods. The opposite is *human* set, in which methods are strongly biased towards negative class giving low sensitivity (less than 70%) and high specificity (almost 100%) which results in unsatisfactory values of *G*_*m*_. The only exception is naïve Bayes which produces results similar to SMOTE + SVM or APLSC.

**Table 3:** Detailed classification results

**Classifier**	**Parameter** **selection (III)**			**Parameter + threshold** **selection (IV)**		
	**SE**	**SP**	***G***_***m***_	**SE**	**SP**	*G*_*m*_
*human*						
N. Bayes	87.98	96.33	92.06	91.97	93.93	92.94
Perceptron	69.56	99.84	83.34	**94.17**	**94.99**	**94.58**
SVM	69.56	99.85	83.34	92.53	95.69	94.10
R. forest	68.21	99.85	82.53	91.53	96.34	93.90
APLSC	94.88	92.14	93.50			
SMOTE + SVM	77.67	99.02	87.69			
*arabidopsis*						
N. Bayes	86.99	98.91	92.76	91.30	97.77	94.48
Perceptron	80.09	99.95	89.47	93.04	97.47	95.23
SVM	80.07	99.96	89.47	93.04	98.95	95.95
R. forest	83.55	99.94	91.38	**95.22**	**99.04**	**97.11**
APLSC	96.09	90.42	93.21			
SMOTE + SVM	88.71	99.64	94.02			
*animal*						
N. Bayes	85.54	95.53	90.40	88.83	92.81	90.79
Perceptron	74.03	99.65	85.89	91.78	95.13	93.44
SVM	72.04	99.74	84.77	90.67	90.09	93.34
R. forest	72.52	99.72	85.04	**92.00**	**95.21**	**93.59**
APLSC	91.93	91.13	91.53			
SMOTE + SVM	84.56	98.68	91.35			
*plant*						
N. Bayes	83.56	97.56	90.29	87.48	95.84	91.57
Perceptron	77.30	99.80	87.83	89.64	97.38	93.43
SVM	73.07	99.85	85.42	89.46	97.93	93.60
R. forest	78.41	99.81	88.47	**90.65**	**97.96**	**94.24**
APLSC	92.77	89.39	91.07			
SMOTE + SVM	81.31	99.32	89.86			
*virus*						
N. Bayes	93.21	93.21	93.21	95.74	92.37	94.04
Perceptron	87.77	98.10	92.79	94.08	95.71	94.89
SVM	90.31	98.10	94.12	**95.38**	**95.35**	**95.37**
R. forest	88.59	98.45	93.39	93.26	96.31	94.77
APLSC	96.61	92.97	94.77			
SMOTE + SVM	91.99	97.14	94.53			
*microPred*						
N. Bayes	80.32	94.27	87.02	89.43	87.91	88.67
Perceptron	82.35	99.37	90.46	90.74	94.65	92.67
SVM	79.31	99.72	88.93	89.29	97.01	93.07
R. forest	75.83	99.66	86.94	**91.89**	**96.36**	**94.10**
APLSC	91.45	90.96	91.21			
SMOTE + SVM	87.70	98.83	93.10			

Applying ROC-select procedure to traditional classifiers (variant IV) balances their sensitivity and specificity significantly improving *G*_*m*_ values (except for naïve Bayes in which gains are moderate). The best results were on average obtained for random forest which beats SMOTE + SVM and APLSC in all datasets. However, multilayer perceptron and SVM also overperformed imbalance-suited methods in the majority of cases. The conclusion is twofold: (1) score function returned by examined classifiers properly ranks instances with respect to the conditional class probability, (2) ROC-select procedure successfully applies this knowledge to solve imbalanced classification problem.

Another interesting observation comes from comparison of imbalance-suited strategies, that is SMOTE + SVM and APLSC. Our experiments confirm previous findings that APLSC is superior to SMOTE [32]. It is especially visible in large and highly imbalanced sets like *human* or *plant*. We explain this by the fact that SMOTE is able to produce only a limited number of informative examples. Above some threshold value, synthetically generated instances introduce only noise. An important observation is that APLSC seems to be the only classifier which is biased towards minority class (sensitivity is always higher than specificity) which may be a useful feature in some applications.

If one analyses absolute results for particular datasets, it becomes clear that animal sets (*human* and *animal*) are more resilient to classification than plant sets (*arabidopsis* and *plant*), even though they are more balanced. This is probably caused by the fact that plant miRNAs are better separated from non-miRNAs in the attribute space, hence they are easier to distinguish. The worst absolute results in terms of *G*_*m*_ were observed for *microPred* dataset. We explain this by the low quality of this set (miRBase 12 was known to contain some false positives removed in later releases [18]) and lack of experimental evidence-based filtering.

### Statistical analysis

**Figure 1:**
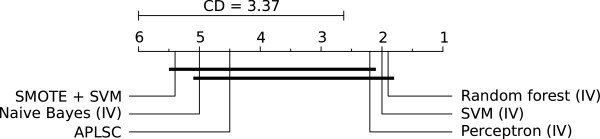
**Statistical significance diagram.** Critical difference diagram for Nemenyi tests performed on *human, animal, arabidopsis, plant, virus* datasets. Average ranks of examined methods are presented. Bold lines indicate groups of classifiers which are not significantly different (their average ranks differ by less than CD value).

In order to statistically evaluate differences between classifiers, Friedman rank test [35] at significance level *α*=0.05 was carried out with *G*_*m*_ being chosen as a performance metric. All the datasets beside *microPred* were used in the procedure. We tested imbalance-suited methods (SVM + SMOTE, APLSC) together with naïve Bayes, perceptron, SVM and random forest in variant IV. The resulting critical difference (CD) diagram for post-hoc Nemenyi tests [35] is shown in Figure 1. As one can see, random forest, SVM and perceptron (which are gathered near rank 2.) outperform APLSC, naïve Bayes and SVM + SMOTE (clustered near rank 5.). Random forest and SVM + SMOTE were confirmed to be the most and least accurate classifiers, respectively. The difference between them as well as the difference between SVM + SMOTE and the second best classifier (SVM) are statistically significant.

### Running time

Time of analysis is an important issue determining applicability of presented methods for real-life problems. As all investigated algorithms are eager learning strategies, testing time was always irrelevant with respect to the training time and is not considered here. In Table 4 medians of training times of all CV runs are given. We show results for the *microPred* set as it was used in other studies, together with *arabidopsis* (the most imbalanced set), *plant* and *animal* (two largest sets). Execution times of most time consuming algorithm variants (IV for naïve Bayes, perceptron, SVM, random forest and III for SMOTE + SVM and APLSC) are given. As all the algorithms were implemented in a serial manner, single analysis utilised just one core of quad-core Intel Xeon W3550 3.06 GHz CPU used for the experiment.

**Table 4:** Training times

**Classifier**	***MicroPred***	***Arabidopsis***	***Plant***	***Animal***
Naïve Bayes	00:00:13	00:01:03	00:06:38	00:11:56
Perceptron	00:28:02	01:15:53	05:15:04	10:21:05
SVM	00:23:00	00:25:49	20:22:57	170:47:13
Random forests	00:17:27	00:59:15	07:58:10	23:07:23
SMOTE + SVM	01:26:00	04:05:17	252:02:10	281:11:12
APLSC	00:00:34	00:01:46	00:08:52	00:29:52

Classifier training times for selected datasets (medians over all cross-validation folds). Times are given in format *hh:mm:ss*.

One should remember that training times are influenced not only by the classification method itself, but also by the number of points in the parameter space to be analysed in a tuning stage. In the case of naïve Bayes classifier no parameters were tuned, thus it was the fastest classifier in the comparison (training times from seconds to minutes). For other classifiers undergoing ROC-select procedure, 20-25 points were evaluated. For smaller sets, training times obtained by multilayer perceptron, random forest and SVM were similar (tens of minutes). For larger sets support vector machines scaled worse than competitors (a few dozen of hours vs. hours). In the case of SMOTE + SVM strategy, 42 points were checked (except *animal* set in which only 25 points were examined). It is important to keep in mind that original microPred included more exhaustive, thus more time-consuming parameter tuning strategy. Limitation of search space did not prevent SMOTE + SVM from being the slowest strategy in our experiments though. In the case of *plant* and *animal* datasets single training took more than ten days which makes microPred strategy inapplicable for larger problems. In contrast, APLSC classifier (4 points in the parameter space) was very fast.

Eventually, we decided to use random forest combined with ROC-select as a basic strategy in HuntMi package due to its superior classification results and reasonable computation time.

### Additional features

The next part of the experiments was to check how introducing additional features influences classification results. These experiments were carried out for random forest + ROC-select combination, selected earlier as a basic strategy in HuntMi. As Table 5 shows, new features introduced additional information into classification procedure and improved final results. The absolute gain in *G*_*m*_ varied from 0.49 to 2.34. Wilcoxon test [35] performed on all datasets beside *microPred* confirmed predominance of the extended representation with *p*-value equal to 0.0952. For this reason we decided to use seven new features together with twenty one previously introduced to represent sequences in HuntMi package.

**Table 5:** Feature selection results

**Dataset**	**SE**	**SP**	***G***_***m***_
*human*	95.31	97.18	96.24
*arabidopsis*	96.11	99.31	97.70
*animal*	94.92	96.60	95.76
*plant*	92.36	98.38	95.32
*virus*	96.18	95.95	96.06
*microPred*	92.76	96.46	94.59

Classification results obtained by ROC-select + random forest combination for extended representation including seven new features. These are also the final results for HuntMi software.

### Comparison with other tools

The majority of miRNA classification studies focus on *H. sapiens*. As microPred was proven to be the best software in this field at the time of its publication, we decided not to consider its predecessors such as Triplet-SVM, MiPred or miPred in the comparison. The results produced by SMOTE + SVM combination on *microPred* dataset were very similar to those obtained by [17] (*G*_*m*_=93.53), which confirms that our experiments accurately estimate microPred performance. The small discrepancy is probably caused by different splits in cross-validation procedure (microPred used 5-fold CV for testing). HuntMi software gave *G*_*m*_=94.59 (see Table 5), which is a noticeable improvement over microPred. The predominance of HuntMi method over SMOTE + SVM combination employed by microPred holds also for all other sets and is statistically significant. To further test the performance of HuntMi, we prepared a set of animal microRNAs newly introduced in miRBase issues 18-19 and examined it on a classification model trained on the entire *animal* dataset (built upon miRBase 17). The obtained results clearly demonstrate that HuntMi is able to efficiently identify novel microRNAs in animals, achieving the sensitivity of over 90% in 8 out of 11 analysed species (Table 6). At the same time the sensitivity achieved by microPred is considerably lower, exceeding 90% only for *O. latipes*.

**Table 6:** Comparison with other tools: animal species

**Species**	**#Sequences**	**MicroPred**	**HuntMi**
*Bombyx mori*	4	75.00	100.00
*Caenorhabditis elegans*	16	87.50	93.75
*Ciona intestinalis*	19	89.47	73.68
*Homo sapiens*	175	85.14	93.14
*Macaca mulatta*	16	-	81.25
*Mus musculus*	139	64.03	94.96
*Oryzias latipes*	152	94.08	96.05
*Pongo pygmaeus*	54	83.33	94.44
*Rattus norvegicus*	38	76.32	97.37
*Taeniopygia guttata*	23	82.61	91.30
*Tribolium castaneum*	14	64.29	78.57

Classification sensitivity of microPred and HuntMi on animal miRNAs added in miRBase issues 18-19.

Several studies on improving microPred have been carried out. They exploited techniques like sample selection [36] or genetic algorithm-based feature selection [37,38] resulting in very high values of *G*_*m*_ (up to 99). All these methods were, however, evaluated on balanced subsets of *microPred* dataset and some of them suffered from important methodological incoherences like lack of random split of data into training and testing set and, more importantly, inclusion of training sequences in a testing set. Therefore, reported results do not accurately estimate the performance of presented strategies in real miRNA identification problems. In addition, these methods are not available as a ready to use packages.

Another strategy, MiRenSVM [39], employed SVM ensembles for miRNA classification. It was tested on moderately imbalanced dataset (697 human miRNAs, 5 428 pseudo harpins) with 3-fold CV resulting in *G*_*m*_=94.76. This value is very similar to the one obtained by HuntMi on *microPred* dataset which consisted of same positive examples and 50% more negatives. MiRenSVM was also tested on a set of 5 238 animal miRNAs successfully identifying 92.84% of them. As no negative sequences were included, specificity of the method is unknown. In our experiments, HuntMi was examined on a set consisting of 7 053 animal miRNAs and 218 154 pseudo hairpins. It outperformed MiRenSVM giving sensitivity of 94.92% and specificity of 96.60%. As MiRenSVM is not available as a tool, we were not able to compare its performance with HuntMi on miRNAs introduced in latest builds of miRBase.

Separate group of methods specialising in plant microRNA identification has been developed, of which the most recent is PlantMiRNAPred [19]. It joins feature and sample selection strategies to improve SVM classification results. The main dataset used in the research consisted of 1 906 real pre-miRNAs from miRBase 14 and 2 122 non-miRNAs generated by authors. 980 positive and 980 negative examples were selected using proposed sample selection method to train the classifier. Majority of the remaining sequences and 309 new miRNAs from miRBase 15-16 constituted the testing set. Surprisingly, as many as 634 training positives were also added to this set. This, together with lack of random split of data into training and testing sets results in overestimation of classification performance. Despite these incoherences, HuntMi performed smililarly to PlantMiRNAPred. After summing up results from PlantMiRNAPred study we obtained *G*_*m*_=96.91, while HuntMi gave 95.32 and 97.70 on *plant* and *arabidopsis* datasets respectively. To further evaluate performance of HuntMi package in plant microRNA classification, we tested it on miRNAs introduced in 18-19 builds of miRBase. Classification model was trained on the full *plant* dataset (constructed upon miRBase 17). As PlantMiRNAPred permits only for manual submissions of single sequences (service for processing FASTA files malfunctioned at the time of this study) we examined it on species with at most 200 newly introduced miRNAs. The results are presented in Table 7.

**Table 7:** Comparison with other tools: plant species

**Species**	**#Sequences**	**PlantMiRNAPred**	**HuntMi**
*Arabidopsis thaliana*	68	80.88	91.18
*Cucumis melo*	120	90.00	95.00
*Glycine max*	302	-	88.41
*Hordeum vulgare*	45	55.56	35.56
*Malus domestica*	206	88.83	99.51
*Medicago truncatula*	300	-	72.67
*Nicotiana tabacum*	163	84.66	93.25
*Oryza sativa*	169	60.95	69.82
*Populus trichocarpa*	89	89.89	97.75
*Sorghum bicolor*	58	94.83	94.83

Classification sensitivity of PlantMiRNAPred and HuntMi on plant miRNAs added in miRBase issues 18-19. PlantMiRNAPred failed to process some *Arabidopsis thaliana* miRNAs successfully. However, these sequences were treated as properly identified.

Based on obtained results, all the plant species examined by HuntMi can be divided into two groups. In the first group (*A. thaliana, C. melo, G. max, M. domestica, N. tabacum, P. trichocarpa, S. bicolor*) the classification sensitivity varied from 88.41% to 99.51% and is clearly superior to the performance of PlantMiRNAPred. The second group (*H. vulgare, M. truncatula* and *O. sativa*) was characterised by much lower sensitivity (35.56% to 72.67%). Two of the latter species belong to monocotyledons, which could suggest that our tool is inefficient when analysing sequences from this plant group. However, we obtained satisfactory sensitivity for *S. bicolor* (94.64%). This encouraged us to look closer at microRNAs from low-sensitivity group and we discovered that a large fraction of miRNAs in these species do not meet commonly recognised criteria for annotation of plant miRNAs e.g. in the case of osa-MIR5489, osa-MIR5484, hvu-MIR6177, hvu-MIR6182, mtr-MIR5741d and some other miRNAs the mature microRNA lies outside the stem part of the hairpin. Additionally, most of new miRNAs were discovered using deep sequencing approach only, where it is sometimes only one or several reads that support the miRNA (e.g. osa-MIR5527). This data is insufficient to confirm that the miRNA is precisely excised from the stem. Similarly to HuntMi, PlantMiRNAPred produces unsatisfactory results when applied to *H. vulgare* or *O. sativa* miRNAs (sensitivities of 56% and 61%).

To sum up, in majority of cases HuntMi was able to obtain better results than competitors even though it was evaluated on larger and more imbalanced datasets. Experiments on animal and plant miRNAs introduced in releases 18-19 of miRBase confirmed that HuntMi outperforms other tools like microPred and PlantMiRNAPred. There are methods reporting higher *G*_*m*_ values than HuntMi. However, they were all tested on balanced datasets, often with important methodological flaws, which obstructs proper judgement of their performance in real-life tasks. Moreover, none of these methods is available as a ready to use package.

## Conclusions

In this study we present a new machine learning-based miRNA identification package called HuntMi. It exploits ROC-select, a special strategy of thresholding score function output by classifiers, combined with random forest, which we find to produce best classification results. Twenty one features employed by microPred software together with seven new attributes are used as a data representation. The method was tested on large and strongly imbalanced datasets using stratified 10-fold cross-validation procedure. Classifiction performance was further verified on miRNAs newly introduced in latest builds of miRBase. As a result, HuntMi clearly outperforms state-of-the-art miRNA hairpin classification tools like microPred and PlantMiRNAPred without compromising the training time.

HuntMi comes with *G*_*m*_-optimised models for *H. sapiens*, *A. thaliana*, animals, plants and viruses. There is a possibility to train a model on any dataset and subsequently use it in classification analysis. This feature may be useful if one is interested in predicting miRNAs in particular species or in applying different optimization criterion than *G*_*m*_ in ROC-select procedure. Therefore, HuntMi offers the highest flexibility of all existing microRNA classification packages.

## Abbreviations

APLSC: Asymmetric partial least squares classification; CV: Cross-validation; FP: False positive; HMM: Hidden Markov model; MFE: Minimum free energy; ROC: Receiver operating characteristic; SE: Sensitivity; SMOTE: Synthetic minority over-sampling technique; SP: Specificity; SVM: Support vector machine.

## Competing interests

The authors declare that they have no competing interests.

## Authors’ contributions

AG and MWS contributed to the manuscript equally. AG prepared implementation of ROC-select method and performed experiments. MWS designed features used in classification and prepared datasets. Both AG and MWS analysed experimental results and drafted the manuscript. MS and IM revised the manuscript and supported the research from statistical and machine learning (MS) as well as biological (IM) side. All authors read and approved the final manuscript.

## Supplementary Material

Additional file 1 **A file with supplementary tables.** Table S1 summarises animal and plant species and viruses from which non-miRNA sequences were extracted. Click here for file
