# Supplementary material for: HuntMi: an efficient and taxon-specific approach in pre-miRNA identification
Source: BMC Bioinformatics. 2013 Mar 5;14:83. doi: 10.1186/1471-2105-14-83 (PMC3686668; doi:10.1186/1471-2105-14-83)
Supplement: Additional file 1 — A file with supplementary tables. Table S1 summarises animal and plant species and viruses from which non-miRNA sequences were extracted. [file 1471-2105-14-83-S1.pdf]

HuntMi: an efficient and taxon-specific approach in pre-miRNA identification (SUPPLEMENT)

Adam Gudyś*[1] , Michał Wojciech Szcześniak*[2] , Marek Sikora[1] and Izabela Makałowska[2]

[1]Institute of Informatics, Faculty Of Automatic Control, Electronics And Computer Science, Silesian University of Technology, Gliwice, Poland

[2]Laboratory of Bioinformatics, Faculty of Biology, Adam Mickiewicz University, Poznan, Poland

Email: Adam Gudyś*- adam.gudys@polsl.pl; Michał Wojciech Szcześniak*- miszcz@amu.edu.pl;

*Corresponding author

Table 1: Summary for animal, plant and virus negative datasets used in the experiments.

Species/virus	No. of sequences
	animals
Rattus norvegicus	24 834
Drosophila melanogaster	7 748
Mus musculus	26 538
Caenorhabditis elegans	4 906
Pan troglodytes	21 312
Gallus gallus	19 526
Macaca mulatta	19 755
Bos taurus	15 695
Danio rerio	5 841
Monodelphis domestica	71 999
	plants
Glycine max	6 654
Zea mays	31 277
Populus trichocarpa	40 152
Selaginella moellendorffii	2 601
Triticum aestivum	1 388
Vitis vinifera	5 976
Oryza sativa	26 881
	viruses
Tomato curly stunt virus	2
Torque teno virus 1	1
Apricot latent virus	11
Carnation Italian ringspot virus	4
Cucumber leaf spot virus	3
Human rhinovirus C	10
Lettuce yellow mottle virus	12
Maize mosaic virus	17
Porcine epidemic diarrhea virus	27
Rabbit vesivirus	11
Soybean mosaic virus	10
Xenopus laevis endogenous retrovirus Xen1	12
Avian carcinoma virus	3
Hepatitis B virus	4
Human immunodeficiency virus 1	9
Simian foamy virus	12
BK polyomavirus	5
Human adenovirus A	44
Human herpesvirus 1	158
Human papillomavirus - 1	9
Porcine respiratory and reproductive syndrome	4
Peach mosaic virus	6
Shrimp white spot syndrome virus	279
Ovine herpesvirus 2	124
Pineapple bacilliform comosus virus	8
Beet yellows virus	13
Blackberry virus Y	17
Cherry green ring mottle virus	10
Squirrel monkey retrovirus - HLB	6
